# Automated Cell Tracking and Analysis in Phase-Contrast Videos (iTrack4U): Development of Java Software Based on Combined Mean-Shift Processes

**DOI:** 10.1371/journal.pone.0081266

**Published:** 2013-11-27

**Authors:** Fabrice P. Cordelières, Valérie Petit, Mayuko Kumasaka, Olivier Debeir, Véronique Letort, Stuart J. Gallagher, Lionel Larue

**Affiliations:** 1 Institut Curie, CNRS UMR3348, plate-forme IBISA d'imagerie cellulaire et tissulaire, Orsay, France; 2 Institut Curie, CNRS UMR3347, INSERM U1021, Normal and Pathological Development of Melanocytes, Orsay, France; 3 Université Libre de Bruxelles (ULB), Laboratory of Image Synthesis and Analysis (LISA), Faculty of Applied Sciences, Brussels, Belgium; 4 Ecole Centrale Paris, Laboratory of Applied Mathematics, Chatenay-Malabry, France; Aix-Marseille University, France

## Abstract

Cell migration is a key biological process with a role in both physiological and pathological conditions. Locomotion of cells during embryonic development is essential for their correct positioning in the organism; immune cells have to migrate and circulate in response to injury. Failure of cells to migrate or an inappropriate acquisition of migratory capacities can result in severe defects such as altered pigmentation, skull and limb abnormalities during development, and defective wound repair, immunosuppression or tumor dissemination. The ability to accurately analyze and quantify cell migration is important for our understanding of development, homeostasis and disease. *In vitro* cell tracking experiments, using primary or established cell cultures, are often used to study migration as cells can quickly and easily be genetically or chemically manipulated. Images of the cells are acquired at regular time intervals over several hours using microscopes equipped with CCD camera. The locations (x,y,t) of each cell on the recorded sequence of frames then need to be tracked. Manual computer-assisted tracking is the traditional method for analyzing the migratory behavior of cells. However, this processing is extremely tedious and time-consuming. Most existing tracking algorithms require experience in programming languages that are unfamiliar to most biologists. We therefore developed an automated cell tracking program, written in Java, which uses a mean-shift algorithm and *ImageJ* as a library. iTrack4U is a user-friendly software. Compared to manual tracking, it saves considerable amount of time to generate and analyze the variables characterizing cell migration, since they are automatically computed with iTrack4U. Another major interest of iTrack4U is the standardization and the lack of inter-experimenter differences. Finally, iTrack4U is adapted for phase contrast and fluorescent cells.

## Introduction

Cell migration is a key process in development, homeostasis and disease [1]. It is essential during organism development to ensure the correct positioning of cells at the appropriate time. Homeostatic processes requiring cell migration include wound repair and the inflammatory response [2]. A chemoattractant is produced at the site of an injury (for example, a wound or an infection) and, as part of an inflammatory cascade, causes immune cells to migrate in the bloodstream and other cells to move away from the injury. A failure of cells to migrate may result in severe defects (such as altered pigmentation and skull and limb abnormalities during development) or pathological conditions (such as defective wound repair and immunosuppression). Conversely, the inappropriate acquisition of migratory capacities may result in tumor cell dissemination [3]. Accurate analysis and quantification of cell migration is required for a thorough understanding of development, homeostasis and disease.

 The tracking of cell migration requires continuous observation of a live organism or living cells *in vivo* or in culture. *In vitro* cell migration experiments are often carried out with primary or established cell cultures as genetic and chemical manipulations in these systems are fast, and it is possible to place cells in different matrices and chemotactic environments. The three most common migration assays are scratch wound (or wound healing) assays, assays in Boyden chambers (and their derivatives) and individual cell migration assays [4,5]. All these assays require a phase-contrast microscope. They can be performed to evaluate extrinsic (chemical or physical [UV, X-Ray] modifications) or intrinsic variables associated with the studied cells (such as siRNA or expression vectors). The haptotactic response is an example of extrinsic modification and can be studied by seeding cells on different extracellular matrices. 

Scratch wound assays are performed on a confluent culture where cells are subjected to four concomitant processes: (i) cytoskeleton reorganization (mainly of cell polarity and the microtubule organizing center [MTOC] reorganization), (ii) individual cell migration, (iii) collective migration and (iv) cell proliferation. The two migration fronts and free space in between are easily monitored. Scratch wound assays are cheap, easy to perform and simple to analyze (for example, Gallagher and colleagues described Excel macros for this purpose [6]), but care must be taken when drawing conclusions as they evaluate four phenomena at the same time.

A Boyden chamber is a holder with compartments separated by a porous membrane. They are classically used to monitor cells squeezing through the pores of a defined diameter. In order to stimulate cell migration and invasion, the composition of the two compartments can be different. This approach is easily performed and simple to analyze. The output of the experiment is the percentage of cells crossing the membrane at a defined stage. However, Boyden chambers are expensive, difficult to coat uniformly and are restricted to endpoint analysis. Such assay does not bring any information for its rate of locomotion, proliferation or survival. Moreover, chemotaxis may be masked by the natural chemokinesis. Finally, the Boyden chamber results may be affected by factors such as adhesiveness of cells to the filter, tortuosity and size of the pore channels and detachment of cells from the bottom surface [7].

 Single-cell migration assays monitor the behavior of individual cells requiring low cell density seeding. Besides any regular cell culture petri dish or glass bottom dish, different types of culture chambers (Dunn-chemotaxis or perfusion culture chambers) can be used for live-cell imaging. Images of the cells need to be acquired at regular time intervals over a period of several hours, on a phase-contrast microscope equipped with an image acquisition system, to monitor their individual movement. The automation of microscope stages makes it possible to study many replicates during a single experimental run, avoiding the problems of inter-experiment variation. The locations of each cell are tracked on these recorded frame sequences. This allows to gather considerable amounts of information, including the direction of migration, average and instantaneous speeds, maximum speed, pausing times, distance covered, persistence, maximum range and estimates the variability of these quantities within the studied population of cells. 

 Manual tracking is the gold standard technique and requires an observer to click on a reference point within the cell for each frame of the movie, for all cells visible in the movie. There are four main difficulties with this technique. It is difficult even impossible, to define “the” reference of the cell as it should be independent of intracellular movement (such as karyokinesis). Often, the center of the nucleus is used as the reference (or its geometrical center named centroid), but a nucleolus may also be selected. Precision can be a problem as it is important to click on the right pixel. Consistency can also be difficult to achieve as the user must click on the same reference many times: in an experiment with four treatments, a minimum tracking set could correspond to 108,000 clicks (180 frames per cell x 50 cells x 4 treatments x 3 replicates). This is extremely time-consuming (≈30 active hours), with operator fatigue leading to the possibility of inaccurate tracking and repetitive strain injuries such as tendonitis. A fourth difficulty associated with this technique is inconsistency between operators.

 Automated cell tracking systems may offer a promising alternative method. Modern computer systems are powerful enough to process many images and tracking algorithms. However, there are currently few tracking algorithms and computer programs that can perform this task effectively, and even fewer are freely available to biologists [8,9]. Cell detection is based on identifying differences between the object of interest (the cell) and the background. Cells can be labeled with fluorophore or detected by phase-contrast, although it is easier to obtain the differential between a fluorescent cell and a dark background than the different pixel intensities of phase-contrast images. In this respect, it is logical that many software packages require fluorescently labeled cells [8]. These packages very effectively identify fluorescent cells due to a high level of contrast between the cells and the background [8]. However, one must not forget that excitation/emission of light induces phototoxicity that may slow and/or kills cells. Cell tracking by phase-contrast is more difficult to achieve than fluorescent tracking due to the lower contrast between the background and the cells. Moreover, many of the available developed packages for edge detection cannot be readily applied in our context since cell shapes may change rapidly and move into the close vicinity of other cells, rendering edge-detection and shape-matching algorithms ineffective. Finally, specific software, such as MATLAB [10], is not available to most biologists, and they are not familiar with program languages such as C++.

We have addressed the current lack of software options for phase-contrast cell tracking by developing a Java-based cross-platform cell-tracking program. The user interface is simple to navigate and provides a visual display of the results. The tracking process is based on a mean-shift principle, allowing rapid tracking at minimal computational cost.

## Results and Discussion

The mean-shift algorithm to track phase-contrast images includes three phases [11]. A pre-processing phase is applied to each acquired image in order to locally enhance image contrast and normalize/equalize gray level histograms. This ensures a better contrast between the inner part of the cell (darker cell center) and the surrounding region of the cell (bright or white halo). Each cell to be analyzed is then manually selected on the last image of the movie by clicking on each of their centers. Automated backward tracking can then be carried out. 

**Figure 1:**
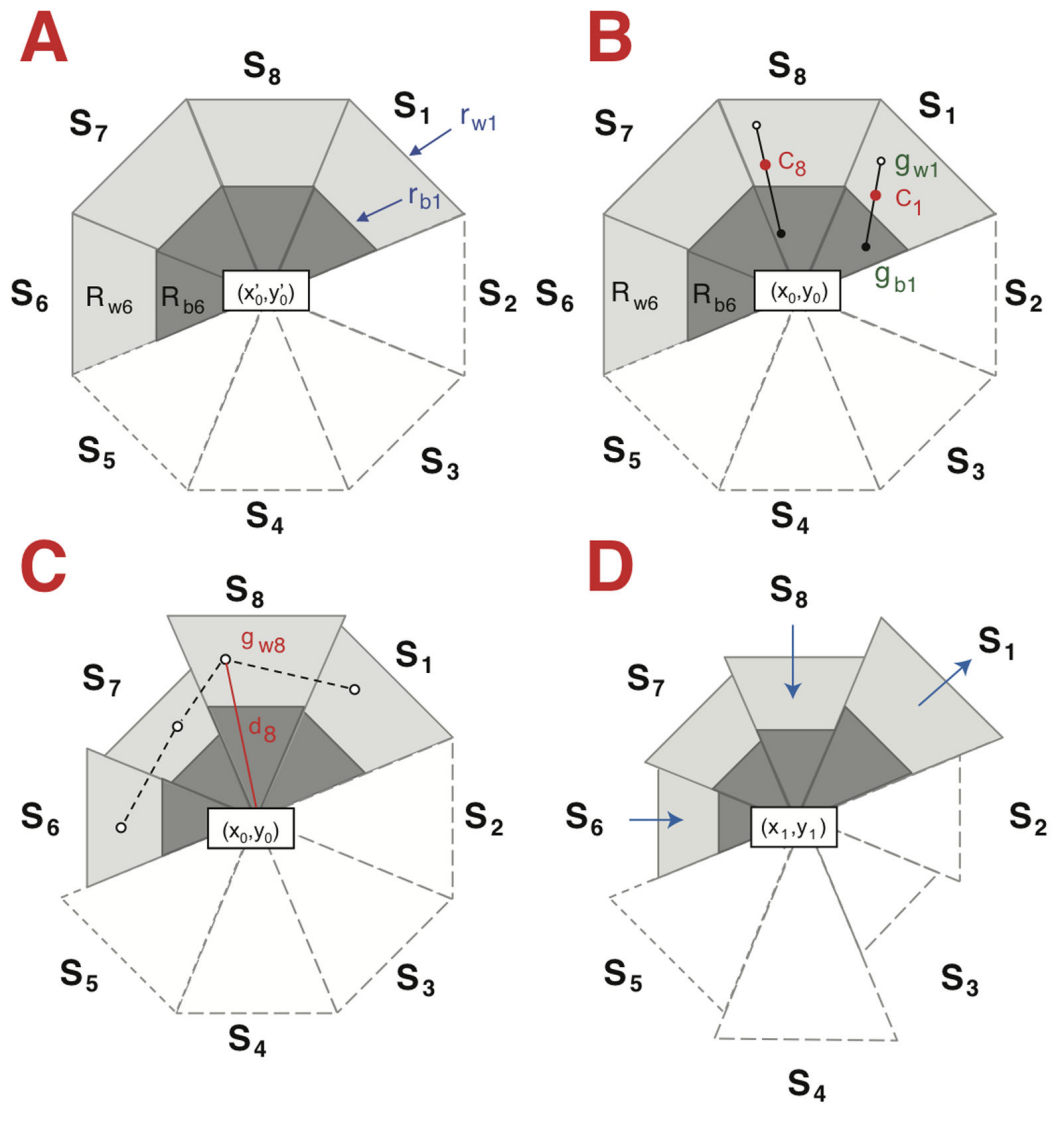
Illustration of the mean-shift model. *A*. Initialization of the generic kernel based on the user-defined position (x’_0_,y’_0_). The kernel (here an octagon, ndir = 8) is divided into sectors (S1 to S8), each one containing two nested triangle-shaped regions (R_b_ and R_w_), one sensitive to dark and the other to bright pixels (“b” means “black” and “w” means “white”). Here, R_b6_ and R_w6_ are shown, with a total of 16 regions (R_b1_ to R_b8_ and R_w1_ to R_w8_). Only the contours of sectors 2-5 are shown in order to lighten and better visualize the figure. The cell is not presented for clarity. *B*. *Adjustment of the position of the center at t_0_*. Sixteen mass centers (g_b1_ to g_b8_ and g_w1_ to g_w8_) are first calculated from the intensities of the pixels from each region, (g_b1_ and g_w1_ are shown). Sector mass centers (C) are calculated from g_wn_ and g_bn_, (C_8_ is shown as an example). The center (x_0_,y_0_) is defined as the centroid of the mass centers C_1_ to C_8_. *C*. *Adaptation of the kernels to cell morphology at t_0_*. The distances (d_i_) between each mass center (g_wn_) and kernel center (x_0_,y_0_) are calculated (d_8_ is shown as an example). The new outer radii (r_wn_) are calculated based on d_n,_ the average d_n_ distances, the expansion factor and the anisotropy factor. r_bn_ is assigned according to the ratio (r_b_ / r_w_), which is initially defined by the user. *D*. *Representation of the kernels at t_1_*. Information is obtained applying the processes explained in B and C. The size of the sectors will increase or decrease (indicated by the arrows) as a function of cell shape modifications.

The algorithm approximates the cell border of each selected cell as a polygon, named region or kernel (Figure 1). This is composed of a defined number of isosceles triangles (‘sectors’) and the tips of all triangles correspond to the center of the cell. Each sector is composed of two nested isosceles triangles. The biggest triangle is attracted by bright pixels and the smallest triangle is attracted by dark pixels, generating novel kernels [11]. 

The algorithm iteratively displaces the kernel from its current position to its next position on the following time frame (backward in time). At origin (t_0_), the cell centroid is at the position (x’_0_,y’_0_), which is defined by the user (Figure 1A). The area around this position is divided into sectors (S). The number of sectors (ndir) is defined by the user and is equal to 8 in this specific case; this generates sectors S1 to S8. Each sector S_n_ is composed of two nested triangle-shaped regions, R_bn_ and R_wn_ (“b” meaning “black” and “w” meaning “white”). At the start, the concatenation of these two series of triangles forms two regular concentric polygons. The radius (height of each triangle) of the outer and inner regions (r_w_ and r_b_) are defined, with r_b_<r_w_. Initially, all r_bn_ radii are equal and all r_wn_ radii are equal for all sectors (Sn)_n=1..ndir_. An adjustment step is performed to relocate the position of the center of the kernel (x_0_,y_0_) (Figure 1B). The mass centers (g_bn_ and g_wn_, 1 ≤ n ≤ ndir) are first calculated from the intensities of the pixels from each region. In this nested context, g_bn_ is attracted towards the dark pixels and g_wn_ is attracted towards the bright pixels, resulting in the detection of two regions; a dark and a bright region. Mass centers Cn of each sector are calculated as the barycenter of g_wn_ and g_bn_ with respective weights P_w_ and P_b_, P_w_ and P_b_, are defined by the user. The center of the polygon is computed as (x_0_,y_0_), the cell center at origin after initialization, by linking all C_n_. The shape and size of generic kernels are adapted to cell morphology (Figure 1C). The distance (d_n_) between each mass center (g_w_) and the kernel center (x_0_,y_0_) is calculated. The new outer radius (r_wn_) for S_i_ depends on an expansion factor (k), an anisotropy factor (β), the mean of the d_n_ distances and d_n_ itself (see Debeir et al., 2005). The inner radius (r_bn_) is directly connected to the outer radius (r_bn_ = γ * r_wn_), γ being a user-defined parameter with 0 ≤ γ ≤ 1. Figure 1D illustrates displacement of each sector and the definition of (x1, y1), the set of coordinates determining the new position of the cell. This process is applied for each time point (t), and for each cell by repeating operation performed in Figure 1B,C. A correct pre-processing, which generate "black objects" surrounded by "white halos", is key to the ability of the algorithm to efficiently follow cells. Under certain conditions, the object can be white and the halo grey. This situation is easily manageable by modifying the weight of the different regions.

We previously validated the automated cell tracking algorithm by comparing this method for *in vitro* analysis of murine melanocytes migration with results generated manually by human experts [12]. Here, we focus on different variables that can be retrieved using this software, and the advantages of Java-based software for biologists who are not familiar with computer programming.

**Figure 2:**
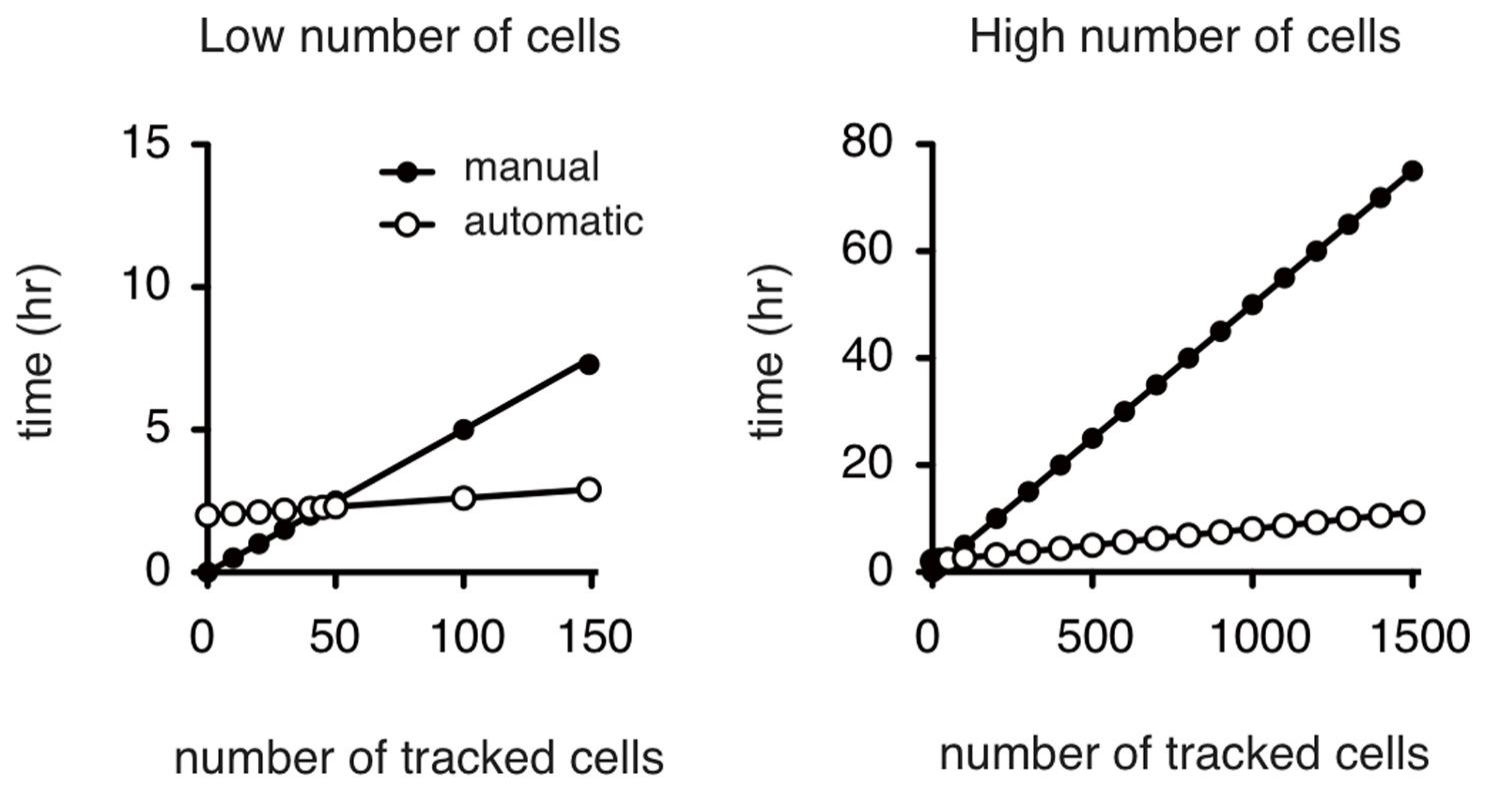
Time benefit of automatic tracking *vs*. manual tracking. Cells were followed either manually (full circles) or using iTrack4U (white circles). It took about three minutes to track each single cell, corresponding to 181 frames, by manual tracking. Twenty cells can therefore be manually tracked in 1 hour and 200 cells during 10 hours of active work. Automatic tracking is performed in two major steps: (i) the establishment of the parameters for both pre-processing and tracking requires about two hours and (ii) the automatic tracking requires about four seconds to fully track a single cell. Using iTrack4U is beneficial for following over about 50 cells.

As detailed in the user's guide (File S1), it takes only a few minutes to pre-process a movie (between 3 and 15 minutes depending on the power of the current processor) when a suitable contrasted sequence of frames is provided (Movies S1 and S2). It then takes less than two minutes to select all the cells of interest to analyze by clicking on them (a 10X objective/0.22 is appropriate for the selection of up to 50 cells), and a few minutes for the software to track the cells (depending on the number of cells selected). The required tracking parameters depend on the type of cell being followed and it can take about two active hours to define these for a specific cell line (Figure 2). Parameters are established using the default parameters and optimized after successive trials. The use of this automatic tracking system becomes beneficial compared to the manual approach as soon as more than 40 to 50 cells of a specific line have to be analyzed.

**Figure 3:**
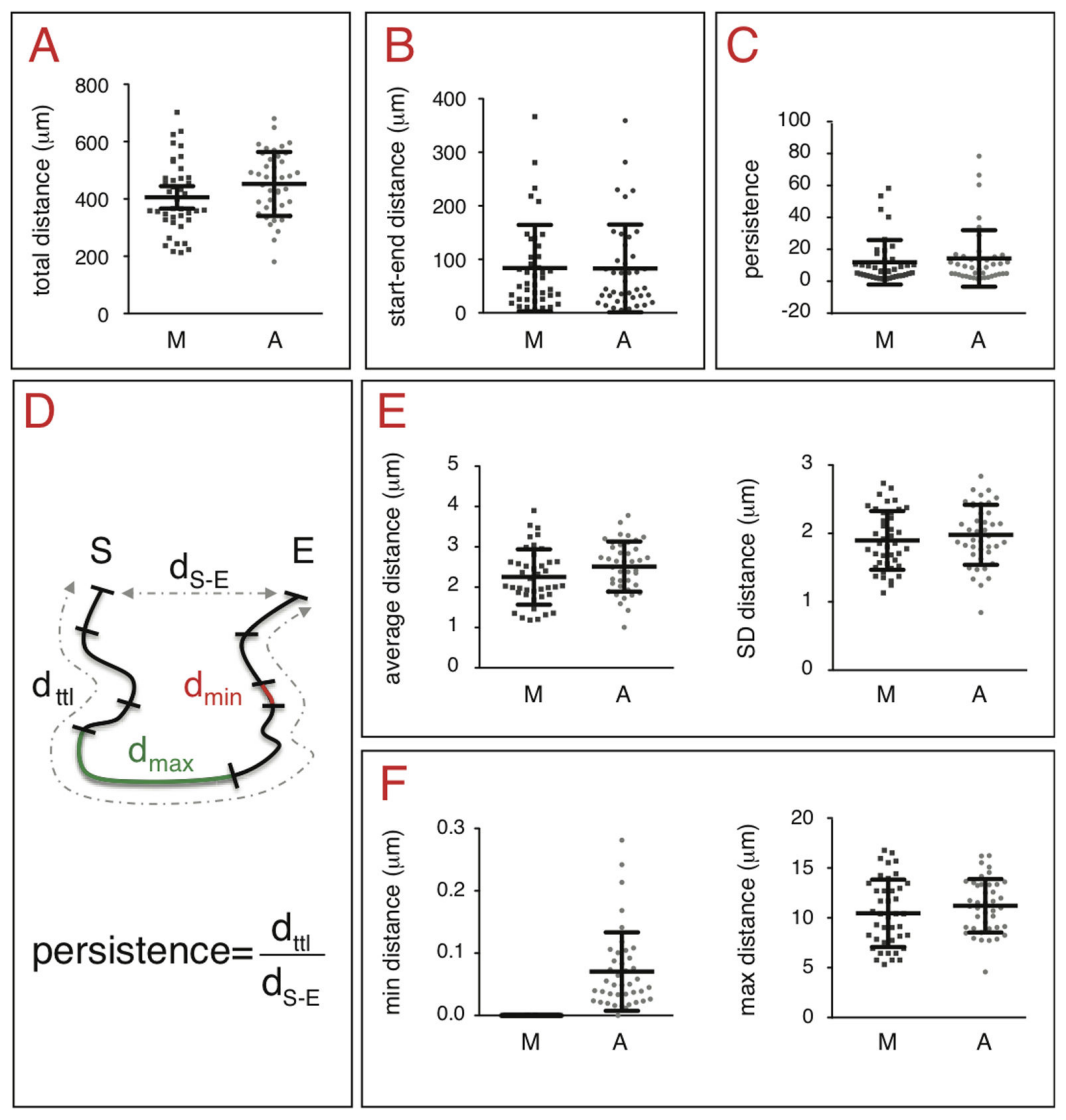
Geometric characteristics of cell trajectories associated with distances and extracted by manual and automatic tracking. Cells were imaged every four minutes for 12 hours and experiments were repeated three times. The same 40 independent cells were tracked manually (M) and automatically (A). The following variables were extracted from the manually and automatically retrieved sets of coordinates:. *A*. *Total distance of migration by WM852 human melanoma cells*. Manual and automatic methods were not statistically significant (standard unpaired t-test, p = 0.0774). *B*. *Euclidian distance (start-end distance) of WM852 human melanoma cells*. Manual and automatic methods were not statistically significant (standard unpaired t-test, p = 0.9672). *C*. *Persistence of migration by WM852 human melanoma cells*. Manual and automatic methods were not statistically significant (standard unpaired t-test, p = 0.5012). *D*. *Definition of migration variables used in this figure*. Total distance = d_ttl_, Euclidian distance = d_S-E_, persistence = d_ttl_ / d_S-E_, minimum travelled distance = d_min_, maximum travelled distance = d_max_. *E*. *Average distance of migration by WM852 human melanoma cells*. Manual and automatic methods were not statistically significant (standard unpaired t-test, p = 0.0774 and p = 0.3913 for the average distance and standard deviation, respectively). *F*. *Extreme values (minimum and maximum distances) of migration for WM852 human melanoma cells*. Manual and automatic methods were not statistically significant for the maximum distance (standard unpaired t-test, p = 0.2611). A significant difference for the minimum distance has no real meaning, as explained in the text (standard unpaired t-test, p = 0.001).

**Figure 4:**
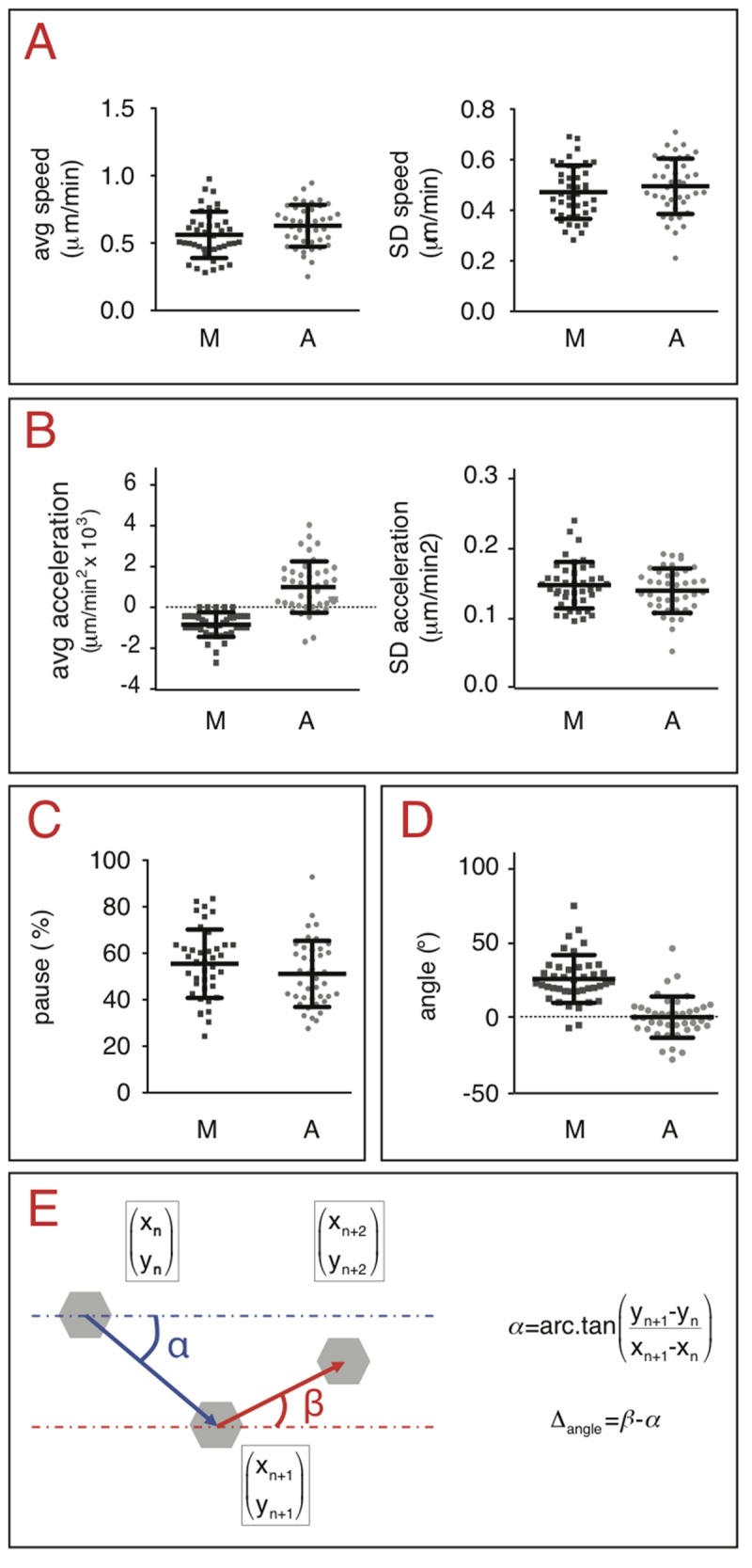
Time-dependent characteristics of cell trajectories extracted by manual and automatic tracking. Cells were imaged every four minutes for 12 hours and experiments were repeated three times. The same forty independent cells were tracked manually (M) and automatically (A). The following variables were extracted from the manually and automatically retrieved sets of coordinates:. *A*. *Average migration speed of WM852 human melanoma cells*. Manual and automatic methods were not statistically significant (standard unpaired t-test, p = 0.0669 and p = 0.3266 for the average speed and standard deviation, respectively. *B*. *Average acceleration of migration by WM852 human melanoma cells*. Manual and automatic methods were statistically significant (standard unpaired t-test comparing average acceleration, p = 10^-4^). For the standard deviation of average acceleration, p = 0.2729. *C*. *Percentage of pause by WM852 human melanoma cells*. Manual and automatic methods were not statistically significant (standard unpaired t-test, p = 0.1783). *D*. *Angles of displacement between two adjacent time frames (angle α) calculated for WM852 human melanoma cells*. Manual and automatic methods were statistically significant (standard unpaired t-test, p = 10^-4^). E. Definition of variables . The polygon (gray hexagon) represents a cell migrating at three different time frames with three sets of coordinates. The angles α and β are defined relative to the horizontal line as a reference at two consecutive times.

Once the sequence of coordinates of a selected cell is computed, the software automatically computes different variables that characterize cell trajectory and motility (as detailed below and in the user's guide [File S1]). To illustrate the results, we analyzed various cell lines, including melanocytes and melanoma, both manually and automatically. The variables describing the migration of the WM852 human melanoma cell line are presented in Figures 3 and 4. The total distance of migration, and the Euclidian distance between the start and end of the track, were evaluated for each WM852 cell and averaged (Figure 3A,B). The persistence of cell migration is defined as the ratio of the total distance over the Euclidian start-end distance (Figure 3C,D). For these variables, the difference between the results extracted by manual and automatic methods was not statistically significant. 

We evaluated distances covered by each WM852 cell between two adjacent frames. The average and standard deviation of these values are then computed over the whole set of frames for each cell. Averages of the above mentioned averages and standard deviations over the cell population were calculated (Figure 3E). There was no significant difference between these variables calculated using manual and automatic methods.

The extreme values (minimum and maximum distances between two consecutive frames) were extracted and averaged (Figure 3F). The maximum distance (approximately 12 μm) was not significantly different between the two tracking methods. Concerning the minimum distance, automatic tracking was evaluated to 0.07 μm (corresponding to 0.05 pixel), whereas the minimum manual tracking distance corresponded to less than 1 pixel. This difference can be explained by some characteristics inherent to each method. Automatic tracking takes into account the intensities in the two sets of kernels to compute the center of the cell. The cell coordinates (x,y), expressed as decimal values, are therefore influenced by both the cell border and the nucleus position. Manual tracking is mainly based on the choice of a reference corresponding to the center of the nucleus or a nucleolus. This reference is unique, its position relies on interpretation by the experimenter, and its coordinates correspond to 1 pixel. Small cell movements are thus detected by automatic tracking using various criteria but not by manual tracking that takes into account only one criterion, the nucleus or nucleoli. Automatic tracking is therefore naturally more sensitive than the manual tracking, which explains the difference between the observed minimal distances.

We also extracted time-dependent and geometric variables including cell speed, acceleration, pause and the angle that a cell generates between two time frames (Figure 4). 

The average speed and its associated standard deviation between two adjacent time frames were evaluated for each WM852 cell. Averages of the above mentioned averages and standard deviations were calculated (Figure 4A); there was no statistical difference between the variables extracted by the two methods.

We calculated the average acceleration between two adjacent time frames (and its standard deviation) for each WM852 cell. Averages of these forty averages and standard deviations were then taken (Figure 4B). The average accelerations were over one hundred times smaller than their standard deviations (0.001 μm/min^2^ *vs.* 0.15 μm/min^2^), which means that this value is highly variable and at the limit of significance. 

The percentage of cell pause during its trajectory was evaluated for each WM852 cell. A cell is considered pausing when the instantaneous speed is below a user-defined threshold (the threshold will depend on cell type). The difference between the percentage of pause extracted from both manual and automatic methods was not statistically significant (Figure 4C).

We evaluated the angle of cell displacement generated between two time frames (Figure 4D,E). Averages of these angles were calculated for all cells (Figure 4D) and we found a statistically significant difference between the results from manual and automatic methods. This can be explained, using the same arguments as previously applied to differences between minimum distances, by the different sensitivity of the two methods to small displacements. 

It is important to take care when analyzing these variables in terms of biological relevance. For example, impressive large maximum distance may indicate that the software has lost a specific cell during tracking. The user can easily visualize the tracks generated by the software to detect most of the inconsistencies that may be observed in the results. Loss of cells is *a priori* not predictable, the cells most commonly lost by the software are those that interact too closely or are going into division.

**Figure 5:**
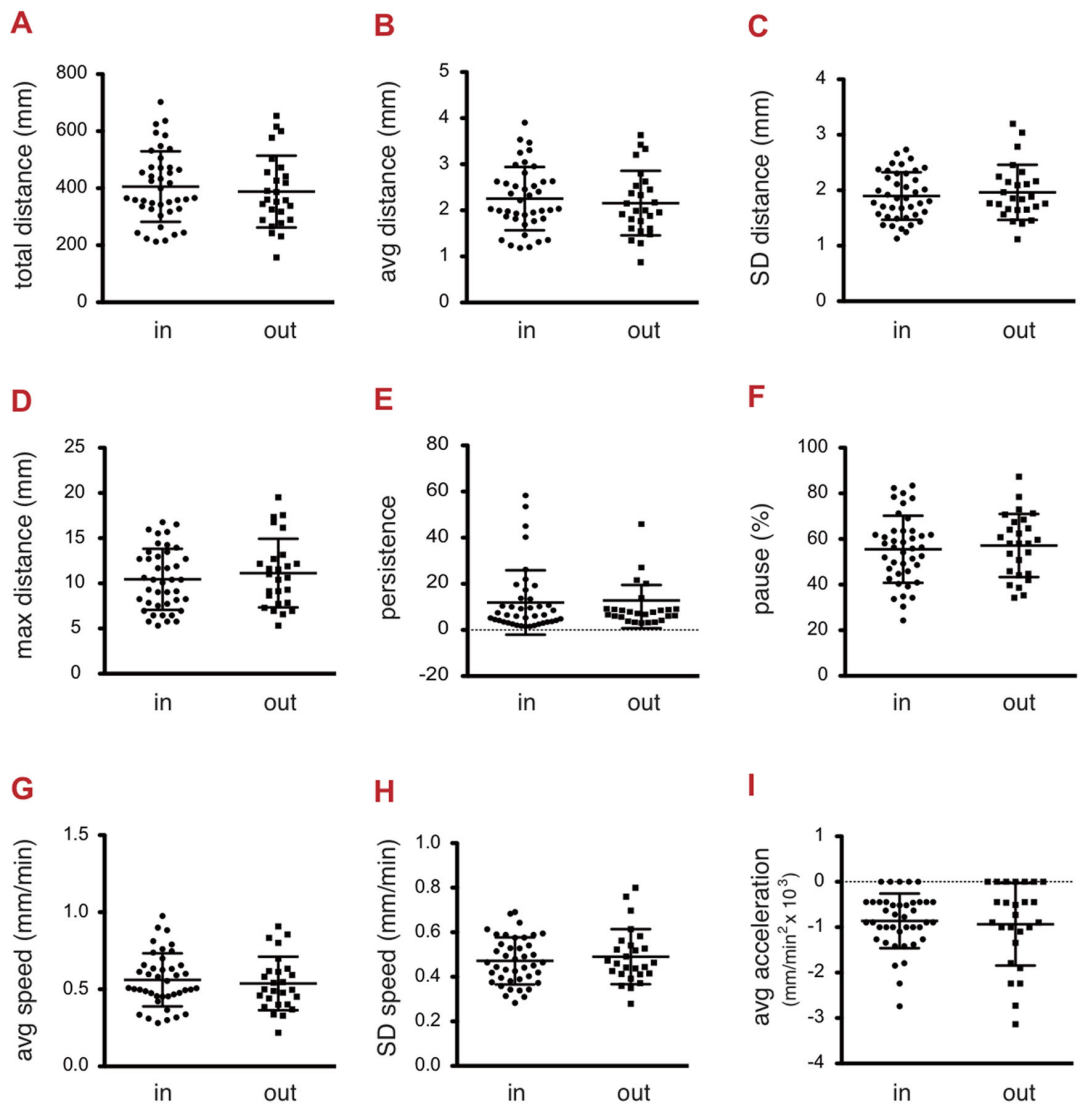
Representativeness of the tracked cells. Cells were imaged every four minutes for 12 hours and experiments were repeated three times. All cells that were lost during the automatic tracking were re-analyzed by the manual method. We compared the following variables for the population of cells followed by the software (41 cells) and the population of cells lost by the software (26 cells), both populations being analyzed manually here. *A*. *Total distance of migration by WM852 human melanoma cells*. The two cell populations did not show any statistically significant difference (standard unpaired t-test, p = 0.5756). *B* and *C*. *Average distance of migration by WM852 human melanoma cells*. The two cell populations did not show any statistically significant difference (standard unpaired t-test, p = 0.5757 and p = 0.5698 for the average distance and standard deviation, respectively). *D*. *Maximum distance of WM852 human melanoma cells*. The two cell populations did not show any statistically significant difference (standard unpaired t-test, p = 0.4433). *E*. *Persistence of migration by WM852 human melanoma cells*. The two cell populations did not show any statistically significant difference (standard unpaired t-test, p = 0.5632). *F*. *Pause of migration by WM852 human melanoma cells*. The two cell populations did not show any statistically significant difference (standard unpaired t-test, p = 0.6459). G and H. *Average migration speed of WM852 human melanoma cells*. The two cell populations did not show any statistically significant difference (standard unpaired t-test, p = 0.5953 and p = 0.5057 for the average speed and standard deviation, respectively). I. *Average acceleration of migration by WM852 human melanoma cells*. The two cell populations did not show any statistically significant difference (standard unpaired t-test, p = 0.6894).

An experimenter manually analyzed two cell populations, (i) the cells successfully analyzed by the automatic method and (ii) those which were not. We found no significant variation between the two cell populations (Figure 5). This series of analyses was performed to rule out any bias in the method, including variations in morphometric aspects, intensity or both.

The software used has a series of advantages but unfortunately also some limitations. The main limitation is its low ability to distinguish between two very close cells, including dividing cells. The algorithm also has very high sensitivity; any modification of cell shape may be considered as a movement, generating exaggerated speed and acceleration. The user must carefully, and retrospectively, analyze the Euclidian distance before drawing conclusions. However, using the software also present numerous advantages. In addition to the considerable amount of time that can be saved when extracting large series of information, other major advantages of automated software include: (i) avoiding bias introduced by different experimenters, (ii) only needing to establish the set of the parameters once (for a given cell type under the same conditions), (iii) automatic calculations of migratory variables, corresponding to the migratory characteristics of this cell line under this specific culture condition, and (iv) the ability to track cells using phase-contrast and/or fluorescence.

## Materials and Methods

### Cell culture

WM852 human melanoma cells were a kind gift from Dr M. Herlyn (Wistar Institute, Philadelphia) and have been previously described [13]. Cells were grown in RPMI supplemented with 10% heat-decomplemented fetal bovine serum (Sigma), 2mM of L-glutamine (Gibco), 100 units/ml of penicillin and 100µg/mL streptomycin (Gibco). Cells were cultured at 37°C, under a humidified atmosphere with 5% CO_2_. 

### Video microscopy

Exponentially growing cells were seeded at a density of 5x10^4^ in a 6-well plate as a regular cell culture petri dish. After 24 hours of incubation, cells were imaged every 4 minutes for 12 hours. All live imaging microscopy was performed on a Leica DM IRB microscope with motorized stage, in a humidified atmosphere of 37°C and 5% CO_2,_ under the control of the Metamorph® software. 

### Migration assay

The cells were followed using the iTrack4U software which is provided as a supplemental file (File S2) and which is also available from https://sites.google.com/site/itrack4usoftware/home .

The nucleus of each cell was manually tracked with the Manual Tracking plugin for *ImageJ* developed by F. Cordelières (http://rsbweb.nih.gov/ij/plugins/track/track.html).

## Supporting Information

File S1 **User's Guide for the iTrack4U software.** The file includes Figure S1 (Interface of the iTrack4U software: the main window and menus), Figure S2 (Example of a phase-contrast movie opened with iTrack4U), Figure S3 (Pre-proc. *Options*), Figure S4 (Preview of pre-processing applied to the first image of the movie), Figure S5 (Setup table), Figure S6 (Manual selection of 10 cells on the last image of the pre-processed movie), Figure S7 (“Detect cells” window), Figure S8 (Empty "Tracking" Table), Figure S9 (Top of the Tracking table, showing for each time points, x and y values), Figure S10 (“Analysis” tab showing characteristics of cell migration), Figure S11 ("Calibration and options" window that allow modification of the conversion pixel/μm, and of the speed threshold ), Figure S12 ("Analysis" tab of the "Full statistical Report" window ), Figure S13 (Path descriptors retrieved from the tracking data ), Figure S14 (*Graphical* *representation* *of* *the* *tracks* *in* *two* dimensions), Figure S15 (3D Graphical representation of tracks), Figure S16 (Centered Graph representation), Figure S17 (Superimposition of the 10 selected cells with their 12 kernels (6 inners & 6 outers) on each frame of the pre-processed movie), Figure S18 (Superimposition of the 10 selected cells with the 6 mass centers on each frame of the pre-processed movie ), Figure S19 (Superimposition of the 10 selected cells with their 12 kernels (inner & outer) and their associated mass centers on each frame of the pre-processed movie ), Figure S20 (Superimposition of a dot representing the center of each cell on each frame of the pre-processed movie ), Figure S21 (Superimposition of a line representing the path of each cell on each frame of the pre-processed movie ), Figure 22 (Dots and Lines are superimposed on the 10 selected cells ) and Figure S23 (“trackVisual…” allows modifications of the dot and line width and font size used to display the cell number).
(DOCX)

File S2 **iTrack4U Software.**
(ZIP)

Movie S1 **Stack of images.**
(ZIP)

Movie S2 **Preprocessed movie.**
(ZIP)
