# Supplementary material for: Automated Cell Tracking and Analysis in Phase-Contrast Videos (iTrack4U): Development of Java Software Based on Combined Mean-Shift Processes
Source: PLoS One. 2013 Nov 27;8(11):e81266. doi: 10.1371/journal.pone.0081266 (PMC3842324; doi:10.1371/journal.pone.0081266)
Supplement: File S1 — User's Guide for the iTrack4U software. The file includes Figure S1 (Interface of the iTrack4U software: the main window and menus), Figure S2 (Example of a phase-contrast movie opened with iTrack4U), Figure S3 (Pre-proc. Options), Figure S4 (Preview of pre-processing applied to the first image of the movie), Figure S5 (Setup table), Figure S6 (Manual selection of 10 cells on the last image of the pre-processed movie), Figure S7 (“Detect cells” window), Figure S8 (Empty "Tracking" Table), Figure S9 (Top of the Tracking table, showing for each time points, x and y values), Figure S10 (“Analysis” tab showing characteristics of cell migration), Figure S11 ("Calibration and options" window that allow modification of the conversion pixel/μm, and of the speed threshold ), Figure S12 ("Analysis" tab of the "Full statistical Report" window ), Figure S13 (Path descriptors retrieved from the tracking data ), Figure S14 (Graphical representation of the tracks in two dimensions), Figure S15 (3D Graphical representation of tracks), Figure S16 (Centered Graph representation), Figure S17 (Superimposition of the 10 selected cells with their 12 kernels (6 inners & 6 outers) on each frame of the pre-processed movie), Figure S18 (Superimposition of the 10 selected cells with the 6 mass centers on each frame of the pre-processed movie ), Figure S19 (Superimposition of the 10 selected cells with their 12 kernels (inner & outer) and their associated mass centers on each frame of the pre-processed movie ), Figure S20 (Superimposition of a dot representing the center of each cell on each frame of the pre-processed movie ), Figure S21 (Superimposition of a line representing the path of each cell on each frame of the pre-processed movie ), Figure 22 (Dots and Lines are superimposed on the 10 selected cells ) and Figure S23 (“trackVisual…” allows modifications of the dot and line width and font size used to display the cell number). (DOCX) [file pone.0081266.s001.docx]

***Automated cell tracking and analysis in phase-contrast videos:***

***Development of Java software based on combined mean-shift processes***

Fabrice P. Cordelières^1,^*, Valérie Petit^2,^*, Mayuko Kumasaka^2^, Olivier Debeir^3^, Véronique Letort^4^, Stuart J. Gallagher^2^ and Lionel Larue^2,$^

^1^Institut Curie, CNRS UMR3348, plateforme IBISA d'imagerie cellulaire et tissulaire, Orsay, France.

^2^Institut Curie, CNRS UMR3347, INSERM U1021, Normal and Pathological Development of Melanocytes, Orsay, France.

^3^Université Libre de Bruxelles (ULB), Laboratory of Image Synthesis and Analysis (LISA), Faculty of Applied Sciences, Brussels, Belgium.

^4^Ecole Centrale Paris, Laboratory of Applied Mathematics, Chatenay-Malabry, France

**Supplemental information**

User Guide v1.0

Table of contents

1. GENERALITIES 4

2. SYSTEM REQUIREMENTS 4

3. GETTING STARTED 5

3a. Prerequisite 5

3b. Launch iTrack4U 5

4. Workflow 6

4a. Start tab 6

4a1. Open 6

4a1a. "Open image" button 6

4a1b. "Open XML" button 7

4a2. Pre-process 7

4a2a. "Pre-process options" and "Pre-process preview" buttons 8

4a2b. "Pre-process launch" button 10

4a3. Batch 10

4a3a. "Batch pre-process" button 10

4a3b. "Full batch process" button 11

4b. “Setup” tab 13

4b1. “Tracking options” button 14

4b2. “Add cell” button 15

4b3. “Detect cells” button 16

4b4. “Show all cells” tick 17

4b5. “Remove all” button 17

4b6. Contextual menu 17

4c. “Tracking” tab 17

4c1. “Track” button 18

4c2. “Remove all” button 19

4d. “Analysis” tab 19

4d1. “Calibration and options” button 20

4d2. “Show full statistical report” button 21

4d2a. “Analysis” tab 21

4d2b. “Summary” tab 22

4d2c. “Graph” tab 22

4d3. “Delete cell” button 26

4e. “Output” tab 26

4e1. Visualization 26

4e1a. “Sectors” button 27

4e1b. “Centers” button 27

4e1c. “Sectors and centers” button 28

4e1d. “Dots” button 29

4e1e. “Lines” button 29

4e1f. “Dots and lines” button 30

4e1g. “Visu. Options” button 30

4e2. “Save Data” 30

4e2a. “Save cells list” button 31

4e2b. “Save tracks list” button 31

4e2c. “Save MT style” button 31

4e2d. “Save analysis” button 31

4e2e. “Save summary” button 31

4e2f. “Save image” button 31

4e2g. “Export to XML” button 31

# 1. GENERALITIES

This tutorial outlines the prerequisites for software use, the setup (parameters) required before carrying out tracking and describes the extracted values (variables). The software is still under development. Please report any bug to the authors (iTrack4U.software@gmail.com).

# 2. SYSTEM REQUIREMENTS

At a minimum, you will need a Java Runtime environment. You can find the latest version at: http://java.com/en/download/index.jsp

You can then run the iTrack4U software under any operating system.

# 3. GETTING STARTED

## 3a. Prerequisite

Make sure that **Java virtual machine** is installed on your computer:

- Open Terminal window and type in java -version, press the return key.

- MacOS: the terminal is found in applications/utilities

- Windows: the terminal is found in start/program/accessories

- Linux: the terminal is accessible with Ctl + Alt + T

- The terminal should display the Java version installed on the computer. Otherwise, download the latest version from the web (<http://java.com/en/download/index.jsp>).

## 3b. Launch iTrack4U

For MacOS, download the "iTrack4U" application and double click on the icon.

For Linux, Windows and alternatives to MacOS, follow steps #1 and #2:

#1 Change to the working directory:

- From the terminal, type in

- Linux and MacOS: cd /path/to/the/software/

- Windows: cd \path\to\the\software\

Alternatively (easier), for all systems

- type in cd (do not forget to add a space after cd)

drag and drop the iTrack4U software folder

press the return key

#2 Start the software and set the maximum available memory for the Java Virtual Machine (JVM):

- From the terminal type in

java -Xmx1599m -jar "iTrack4U.jar"

press the return key

*N.B. ‘1599’ refers to the maximum amount of memory the JVM might use. This parameter should be equal to 75% of the RAM, up to a limit of 1599Mo when using a 32-bit system.*

The interface corresponding to the main software window will pop up (Figure S1).

In the next section, you will be guided through the five steps of the analysis process: “Start”, “Setup”, “Tracking”, “Analysis” and “Output”.

# 4. Workflow

## 4a. Start tab


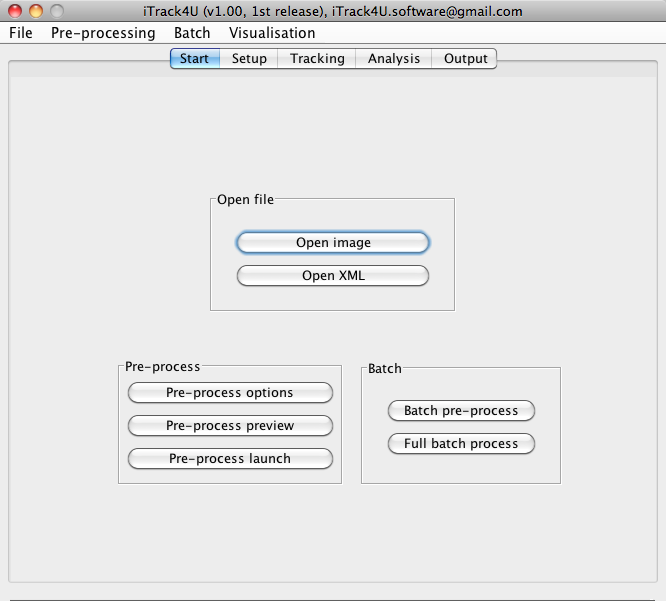


***Figure S1.*** *Interface of the iTrack4U software: the main window and menus.*

The Start tab window has three parts: “Open”, “Pre-process” and “Batch”.

### 4a1. Open

This contains the two options to open a stack of images or open the parameter files as XML files.

#### 4a1a. "Open image" button

Principle: A stack of images is required to perform the tracking. TIFF and TIFF-compliant formats such as STK and ZIP encapsulated TIFF are supported. An example of such stack of images is given as movie S1.

- Click on the button “open image”.

- The navigation window will appear.

- Select the file of interest and click on “open”.

*N.B. 1: Movie navigation (Figure S2) is achieved either using the slider (at the bottom of the window) or using the mouse wheel.*

*N.B. 2: cmd (or ctrl) + and - keys may be used to zoom in and out.*

*N.B. 3: The upper part of the window tells the user the number of the displayed frame, the size of the pictures, the size of the file and its format (Figure S2).*

*Example: Open Movie S1, which is provided as supplemental material.*


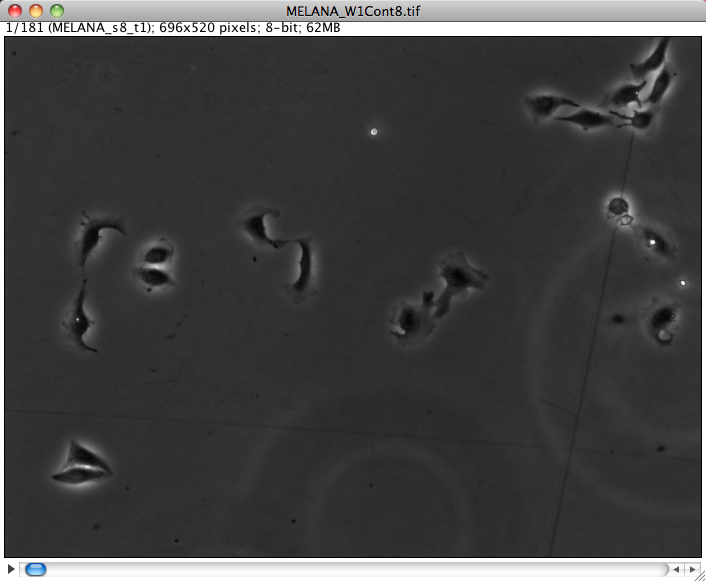


***Figure S2.*** *Example of a phase-contrast movie opened with iTrack4U. The upper part of the window shows the number of the displayed frame (1), the total number of frames (181), the size of the picture in pixels (0 ≤ x < 696 and 0 ≤ y < 520), the format (8-bit) and the size of the movie (62 Mb). On the bottom part of the window, a horizontal scroll bar facilitates visualization of the 181 frames sequence. Note that the quality of the phase-contrast may not be optimal.*

#### 4a1b. "Open XML" button

Principle: The XML file is progressively generated as each step of the analysis process is carried out. It contains: (i) at least, the software options, (ii) once cells for analysis have been selected, their initial coordinates and (iii) once cells have been tracked, their coordinates over time.

- Click on the button “Open XML”.

- The navigation window will appear.

- Select the file of interest and click on open.

*N.B. Each XML file corresponds to an Image file. The Image file has to be opened before the XML file can be opened.*

### 4a2. Pre-process

Principle: Image pre-processing is performed in three steps.

(i) Applying a morphological gradient to the original image allows it to be partitioned into two areas, cell and non-cell, corresponding to high and low contrast areas, respectively. This part of the pre-processing is complete when the threshold values of the mask cover the cells and their halos sufficiently; black pixels correspond to the background and white pixels to the cells.

(ii) A local histogram equalization is performed on the original image to enhance the halo surrounding the cells.

(iii) Images obtained from steps (i) and (ii) are combined to generate the pre-processed image.

#### 4a2a. "Pre-process options" and "Pre-process preview" buttons

- Step 1. Click on the button “Pre-process options”.

1a. The “Pre-proc. options” window will appear (Figure S3).

1b. Set the “Morphological gradient” parameters.

1b1. Tick the “Morphological gradient” and untick the “Local equalization” boxes.

1b2. Start by applying the default parameters (Gradient radius = 12 and Gradient threshold = 50), tick “Preview when clicking on OK”, and click OK to close the window.

1b3. Evaluate the mask on the newly generated image to check if it covers the different cells and their halos.

If no, click on the original image and modify the Gradient radius and/or Gradient threshold values. Repeat 1b2 until you find the best conditions.

If yes, close the image “Preview_GradRad=12ThrRad=50” and go to the following step.

*N.B. 1: A default gradient threshold value of 50 refers to an 8-bit image.*

*N.B. 2: The preview is performed on the active image window, on the first frame of the image sequence.*

*N.B. 3: Note that the parameters and their values are written on the upper part of the window.*

*N.B. 4: For the given example, the parameters are Grad Rad = 12, Thr Rad = 30, Eq Rad = 10*


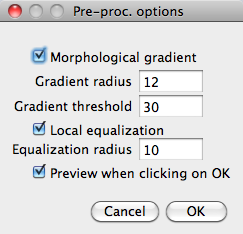


***Figure S3.*** *Pre-proc. options. This dialog box gives access to Morphological gradient, Local equalization and Preview functions.*

- Step 2. Click on the button “Pre-process options”.

1c. The “Pre-proc. options” window will appear.

1d. Set the “Local Equalization” parameters.

1d1. Tick the “Local equalization” and untick the “Morphological gradient” boxes.

1d2. Start by applying the default parameters (equalization radius=10), tick on “Preview when clicking on OK”, and click OK to close the window.

1d3. Evaluate whether the cells are nicely distinguishable with an enhanced halo on the newly generated image.

If no, click on the original image and modify Equalization radius value. Repeat 1d2 until you find the best conditions.

If yes, close the image “Preview_EqRad=10” and go to the following step.

*N.B. 1: The Equalization radius must be less than the Gradient radius.*

- Step 3. Click on the button “Pre-process options”.

1e. The “Pre-proc. options” window will appear.

1f. Set the final parameters for pre-processing.

1f1. Tick the “Morphological gradient”, “Local equalization” and “Preview when clicking on OK” boxes.

1f2. Apply the parameter values defined in steps 1 and 2 and click OK to close the window.

1f3. Evaluate the newly generated image (Figure S4) to check that the mask created in step 1 fits with the shape of the cells with their white halos created in step 2.

If no, fine tune the parameters.

If yes, close the image and go to the following step.


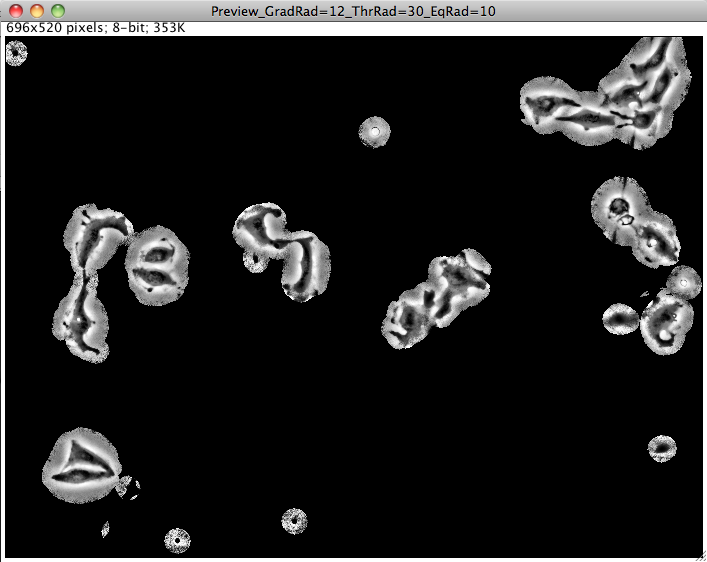


***Figure S4.*** *Preview of pre-processing applied to the first image of the movie. The values of the pre-processing parameters are displayed at the top of the window.*

#### 4a2b. "Pre-process launch" button

Principle: A pre-process preview is performed on the first image. In this step, the entire movie is processed. Make sure that the original movie corresponds to the active window. The preprocessed movie is available as movie S2.

- Click on the button “Pre-process launch”.

*N.B. 1: You can follow the processing on your terminal window. The number of slices corresponds to the number of frames.*

*N.B 2: The pre-processing course may take few minutes to complete; depending on your computer and its configuration it may take between 20 seconds and four minutes.*

*N.B 3: It is recommended to save the pre-process stack and associated data in the “Output” tab*.

### 4a3. Batch

Batch processing allows a defined series of operations to be performed on a set of images. Using iTrack4U, batch processing can be applied to either pre-process a dataset made of several movies or to fully automate a pre-processing/tracking/analysis process.

#### 4a3a. "Batch pre-process" button

- 1. Click on the button “Batch pre-process”.

- 2. A file selection window will appear, labeled “Point to the input directory”.

- 3. Choose the folder containing all the movies that should be pre-processed and select one of the images.

- 4. Click on “Open”.

- 5. A second file selection window will appear, labeled “Point to the output directory”.

- 6. Choose the folder where the pre-processed movies should be saved.

- 7. Click on “Save” to validate your choice.

- 8. The “Pre-process options” window will appear: use the appropriate parameters defined following steps described in paragraph §4a2a.

- 9. Click on “Ok” to start the successive pre-processing of all the files contained in the input folder.

#### 4a3b. "Full batch process" button

**WARNING**: Use of this function requires the user to be familiar with the iTrack4U software. Please read the rest of the full manual, avoiding this section, before coming back to this topic.

- Step 1. Define cells that should be tracked.

*For each movie:*

1a. Open the image (see paragraph 4a1a)

1b. Define the pre-processing parameters, using the “Pre-process options” button (see paragraph 4a2a).

1c. Go to the “Setup” tab (see paragraph 4b).

1d. Choose the right tracking parameters (see paragraph 4b1).

1e. Add cells to be tracked (see paragraph 4b2).

1f. Go to the “Output” tab (see paragraph 4e).

1g. Save the data as an XML file (see paragraph 4e2g).

- Step 2. Set the relevant input/output folders.

2a. Go to the “Start” tab.

2b. Press the “Full batch process” button.

2c. A file selection window will appear, labeled “Point to the input directory”.

2d. Choose the folder containing all the movies that should be fully processed.

2e. Click on “Save” to validate.

2f. A second file selection window will appear, labeled “Point to the input XML directory”.

2g. Choose the folder containing all the XML files that should be used for processing (one per movie to analyze).

2h. Click on “Save” to validate.

2i. A third file selection window will appear, labeled “Point to the output directory”.

2j. Choose the folder where the pre-processed movies and further tracking analysis files should be saved.

2k. Click on “Save” to validate.

- Step 3. Define the output visualization options.

3a. A window will appear where options can be set:

- “Do pre-processing”: tick this to perform the pre-processing.

- “Save results as a stack”: choose the desired option from the list (see paragraph 4e1 for a detailed description of the available options).

- “Save results as a plot”: choose the desired option from the list (see paragraph 4d2c for a detailed description of the available options).

- 3b. Click on “Ok” to start the successive full processing of all files contained in the input folder.

## 4b. “Setup” tab

The ”Setup” tab is used to prepare the tracking process (Figure S5a). The “Tracking options” button is used to select default parameters or modify these options. Cells then need to be selected either manually (“Add cell” button) or automatically (“Detect cells” button).


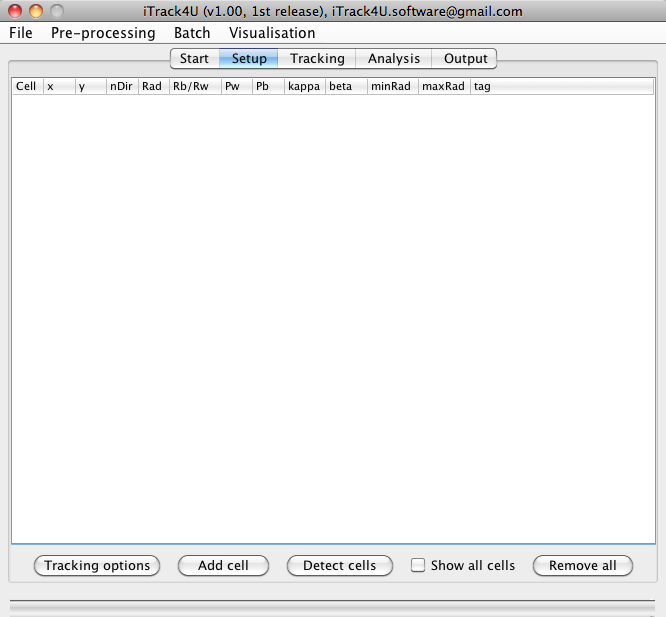
a)

b)

**
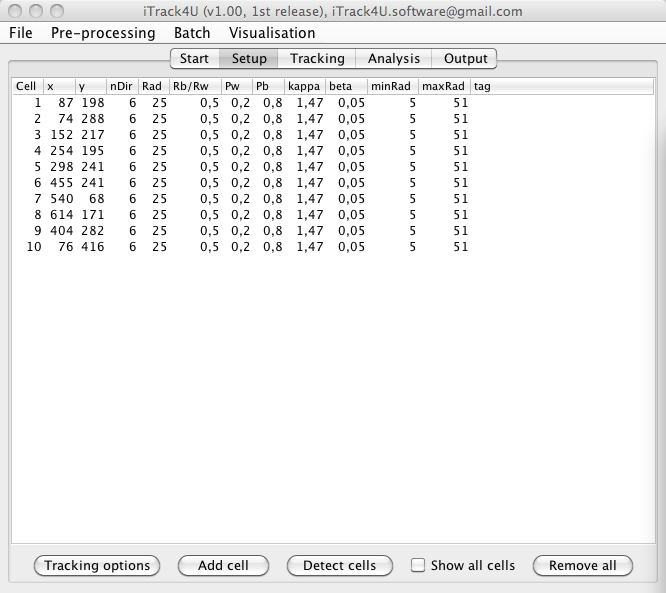
**

***Figure S5.*** *Setup table. a) An empty setup table. b) A table listing 10 selected cells and their associated parameters. x and y correspond to the original x_0_ and y_0_ coordinates of the cells.*

### 4b1. “Tracking options” button

Click on the "Tracking options" button to open a new window. Predefining a series of parameters is essential to optimize the tracking efficiency. This step can be time consuming when starting to analyze a novel cell line. Seven parameters and two sets of options need to be determined:

Parameters

- **Number of kernel sectors** (default: 8) defines the number of sectors used to describe the cell’s outline. A compromise has to be found: a larger number of sectors will help refine the cell’s contours but will lower the number of pixels used to place/move the sectors and reduce the accuracy of cell center localization.
- **Radius of the outer kernel** (default: 25) roughly corresponds to the cell’s radius.
- **rb/rw ratio** (default: 0.50) will influence the position of the boundary between bright and dark areas within each sector.
- **Weight for Kw = Pw** (default: 0.20) **and weight for Kb = Pw** (default: 0.80) with Pw + Pb = 1. Both values define the influence of the bright and the dark areas on the center position of sectors. The influence of Pb or Pw is directly proportional to their given value. This is also a way to compensate for fewer pixels in the black area than in the the halo/bright area, modulating the overall intensities in each area.

*N.B. When you follow fluorescent cells, Pw = 1 and Pb = 0.*

- **Model expansion factor** (default: 1.55) is used to avoid overall kernel collapse as the kernel at the next time point is computed from the centers of sectors centers at the previous time point.
- **Anisotropy level** (default: 0.05) corresponds to the degrees of freedom for modifications of sectors. When the value is high (close to 1) the radii of all sectors are similar. For elongated cells, it is more appropriate to use a value corresponding to the default value.

Options

- **Stop tracking**

Limits are defined to stop tracking when inappropriate minimum and maximum outer radius values are defined. Tracking is stopped if rw is below 5 or above 51 (as default values).

*N.B. When mis-tracking occurs, a kernel might jump from one cell to its neighbor or encompass an adjacent cell. As a result, the kernel might shrink or enlarge. A user may wish to define an interval of truthful values outside of which tracking could be canceled.*

- **Number of trials**

Number of trials to find the center of a kernel (default: 5): the number of times the mean-shift step should be applied to the nested sectors, for each time point.

Number of trials for adapting a kernel (default: 5): the number of times the mean-shift step should be applied to the nested kernels, for each time point.

Once the parameters and options are set, click “OK” and you will be directed back on the “setup” tab.

### 4b2. “Add cell” button

Principle: Cells of interest can be manually selected and their position stored in the setup table (Figure S5b).

- Click on the “Add cell” button to activate the cell selection mode.
- Click on each single cell of interest. Each click generates a new line in the table (including coordinates [x, y] and parameters [such as nDir, Rad and Rb/Rw]). An example of 10 selected cells is shown in Figure S6.
- To exit the cell selection mode, either click on the “Stop add” button or click on another tab.

*N.B. 1: Ticking the “Show all cells” box allows the images of the cells that have been selected to be visualized.*

*N.B. 2: After clicking on the "Add cell" button, the software goes directly to the last frame of the movie as the software tracks the cells in a backward direction.*


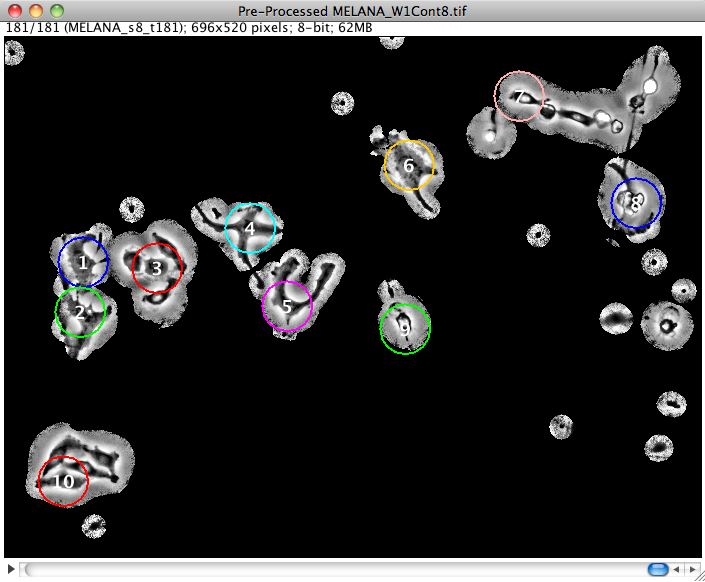


***Figure S6.*** *Manual selection of 10 cells on the last image of the pre-processed movie (by clicking on each cell). Each cell is assigned a number from 1 to 10, (number in white, circled in color). The size of the radius is defined by the options selected for the tracking parameters.*

### 4b3. “Detect cells” button

Principle: Cells of interest can be automatically selected based on their intensities and areas and their position stored in a table (Figure S5).


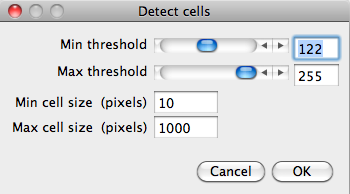


***Figure S7.*** *“Detect cells” window*

- Click on the “Detect cells” button.

- The “Detect cells” window will appear (Figure S7).

The relevant window of intensity is defined using the two sliders.

Filtering based on cell size is achieved by modifying the minimum and maximum values.

*N.B.: This option is very convenient for fluorescent cells but is more delicate when using phase-contrast.*

### 4b4. “Show all cells” tick

By ticking “show all cells”, the initial position of all selected cells can be visualized on the Pre-process movie.

By unticking “show all cells”, you do not see the initial position of all selected cells on the pre-process movie but all information remains in the Setup table. A single cell can be visualized by clicking on the cell/line of interest in this Setup table.

### 4b5. “Remove all” button

This deletes all selected cells from the Setup table.

### 4b6. Contextual menu

Other options are made available on the Setup table using the right click of your mouse.

- **Edit cell**: this opens a “Update cell’s parameters” window and allows parameters to be edited directly.
- **Delete cell**: this deletes a specific cell.
- **Apply current settings to all cells**:

After manually modifying parameters for one cell, this enables the user to apply this modification to all cells of the list.

- **Renumber cells**: when deleting one cell from the full list the cell numbering is unchanged. This option allows cells to be renumbered using a continuous set.
- **Test parameters**: this generates several copies of the currently selected cell (or of all cells) with a single parameter varying between two values which the user defines.
- **Set current parameters to default**: this makes the current parameters the default values for any newly added cell.

## 4c. “Tracking” tab

The “Tracking” tab launches the tracking process. Clicking on this tab opens the tracking window that was originally empty (Figure S8).

In this window there are two options: (i) launch cell tracking and (ii) delete tracking data.


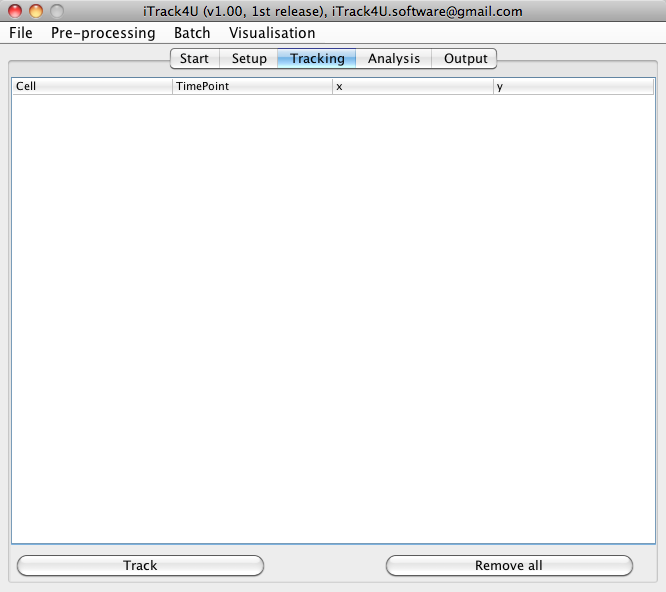


***Figure S8****: Empty "Tracking" Table.*

### 4c1. “Track” button

Click on this button to start the tracking process. At the end of tracking, the table containing cell numbers, time points, and x and y values will be filled (Figure S9).

*N.B. Clicking on any line reveals the outer kernel of the cell of interest at a specific time point.*


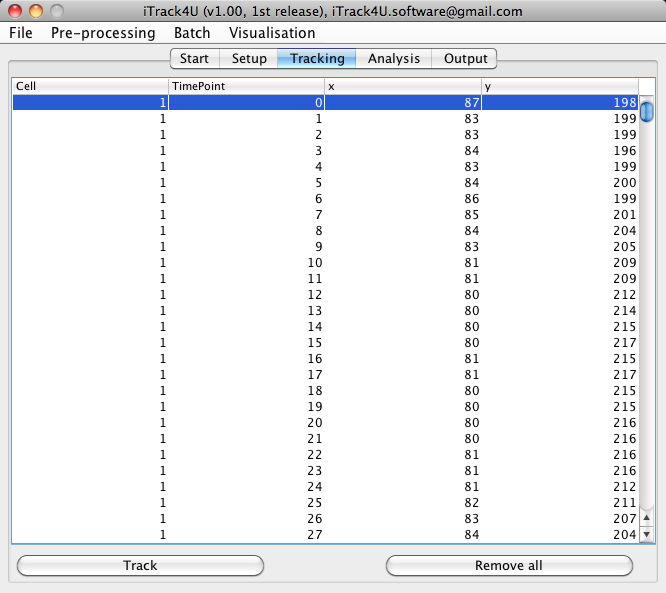


***Figure S9****: Top of the Tracking table, showing for each time points, x and y values. The vertical scroll bar on the right of the window allows visualization of the entire Tracking table for each cell that has been tracked.*

### 4c2. “Remove all” button

This deletes the tracking data from the table.

## 4d. “Analysis” tab

The “Analysis” tab enables all the computed data generated by tracking to be viewed. Clicking on this tab opens the analysis window that is originally filled with a selection of results. These include the cell number, the number of frames which were successfully tracked, including the average speed and the maximum distance (Figure S10).

In this window there are two options: (i) launch cell tracking and (ii) delete the tracking data.


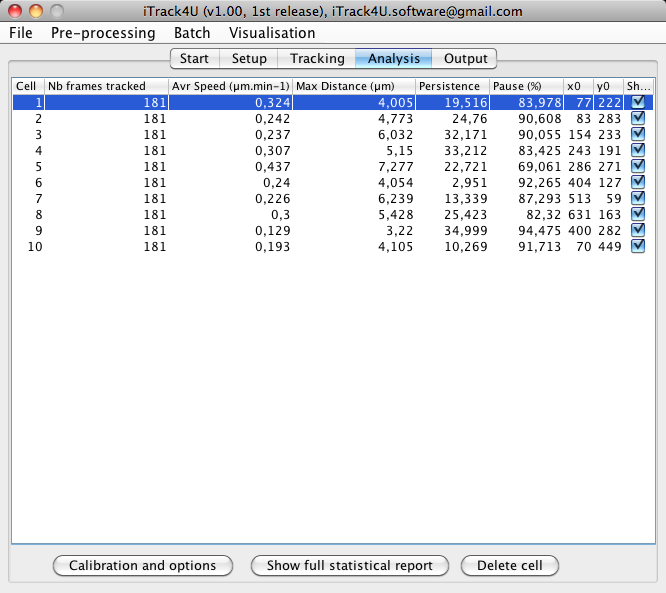


***Figure S10.*** *“Analysis” tab showing characteristics of cell migration. This gives access to the calibration options, shows a full statistical report and enables cell deletion from the list.*

### 4d1. “Calibration and options” button

This aims to identify and modify conversion factors to allow all retrieved

es to be expressed in physical units (μm). By clicking on the “Calibration and options” button a new window appears (Figure S11). It contains:

- x and y calibrations. These values depend on your microscope set up (ask your imaging facility manager).

- time interval, corresponding to the time between two frames.

- speed threshold, corresponding to the minimum speed for which the cell is not pausing.


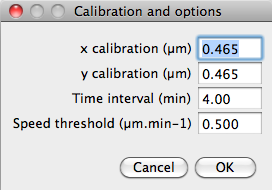


***Figure S11****: "Calibration and options" window that allow modification of the conversion pixel/μm, and of the speed threshold.*

### 4d2. “Show full statistical report” button

By clicking on "Show full statistical report" on the bottom of the window, a new window containing four tabs (“Analysis”, “Summary”, “Graph” and “Filter”) will open (Figure S12).


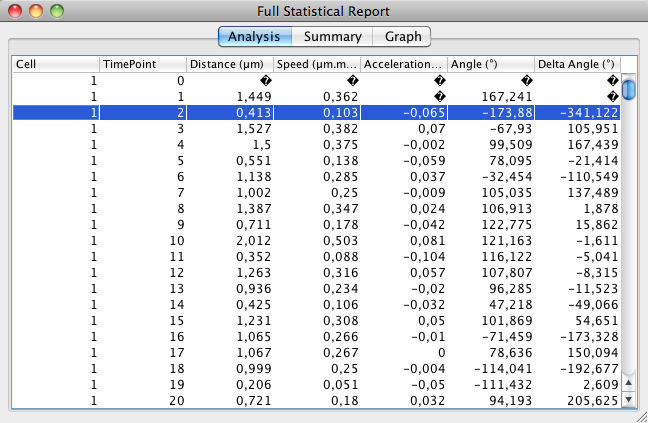


***Figure S12:*** *"Analysis" tab of the "Full statistical Report" window.*

#### 4d2a. “Analysis” tab

The “Analysis” tab gives access to instantaneous information about each cell between two consecutive time points. This includes the distance (μm), the speed (μm/min), the acceleration (μm/min^2^), the angle (°) and the delta angle (Figure S12).

**Distance** (μm): Euclidian distance between two consecutive time points (also known as instantaneous distance).

**Speed** (μm/min): Instantaneous distance divided by time interval (also known as instantaneous speed).

**Acceleration** (μm/min^2^): Difference between two consecutive instantaneous speeds divided by the time interval (also known as instantaneous acceleration).

**Angle** (°): Angle of displacement relative to the horizontal line.

**Delta Angle** (°): Differences between two consecutive instantaneous angles.

#### 4d2b. “Summary” tab

The Summary tab gives access to the full statistical report for each cell and its path and contains information related to distances, time and angles (Figure S13).

**Nb frames tracked.** The number of frames successfully followed by “ITrack4U”.

**Avr Distance** (μm): average instantaneous distance.

**SD Distance** (μm): standard deviation of instantaneous distances.

**Min** **Distance** (μm): minimum instantaneous distance.

**Max** **Distance** (μm): maximum instantaneous distance.

**Total** **Distance** (μm): total distance from t_0_ and t_end_.

**Start-End** **Distance** (μm): the Euclidian distance travelled by the cell between t_0_ and t_end_.

**Persistence** (no unit): ratio of the total distance and the start-end distance.

**Pause** (%): see definition and threshold described in section 4d1.

**Avr Speed** (μm/min): average instantaneous speed.

**SD Speed** (μm/min): standard deviation of instantaneous speeds.

**Avr Acceleration** (μm/min^2^): average instantaneous acceleration.

**SD Acceleration** (μm/min^2^): standard deviation of instantaneous acceleration.

**Avr Angle** (°): average instantaneous angle.

**SD Angle** (°): standard deviation of instantaneous angles.

**Avr Delta Angle** (°): Average instantaneous delta angle.

**SD Delta Angle** (°): standard deviation of instantaneous delta angles.


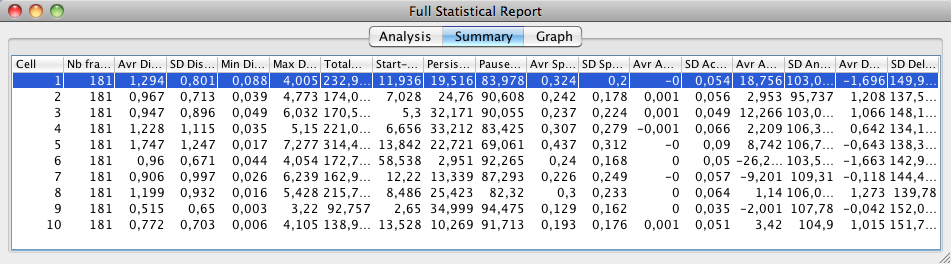


***Figure S13.*** *Path descriptors retrieved from the tracking data. This table is generated from the analysis tab of the full statistical report.*

#### 4d2c. “Graph” tab

The Graph tab gives access to various graphical representations of the tracking paths. By default, a 2D representation is shown (Figure S14).


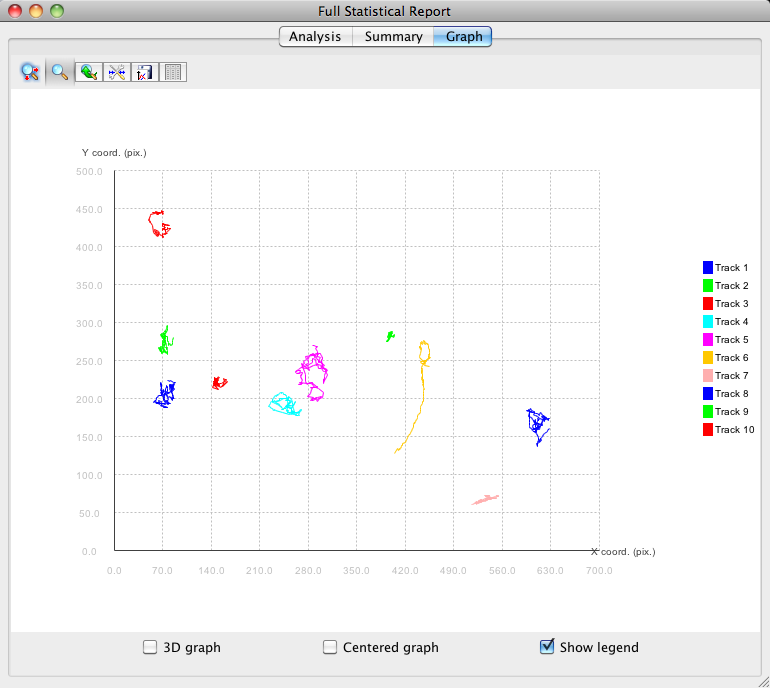


***Figure S14.*** *Graphical representation of the tracks in two dimensions (x,y). These graphs are generated from the graph tab of the full statistical report.*

Three tick box options enable or disable the following variables:

**3D graph** (Figure S15): switches from the 2D view (x,y) to the 3D view (x,y,t). The 3D graph is interactive (click and drag).


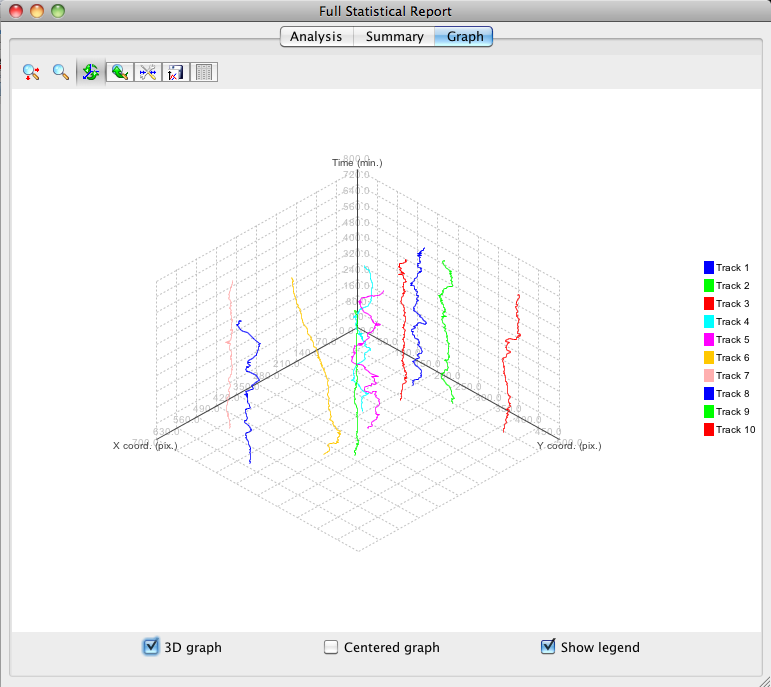


***Figure S15****. 3D Graphical representation of tracks.*

**Centered graph:** (Figure S16) If ticked, this view moves all tracks back to the same origin (in both 2D and 3D options).


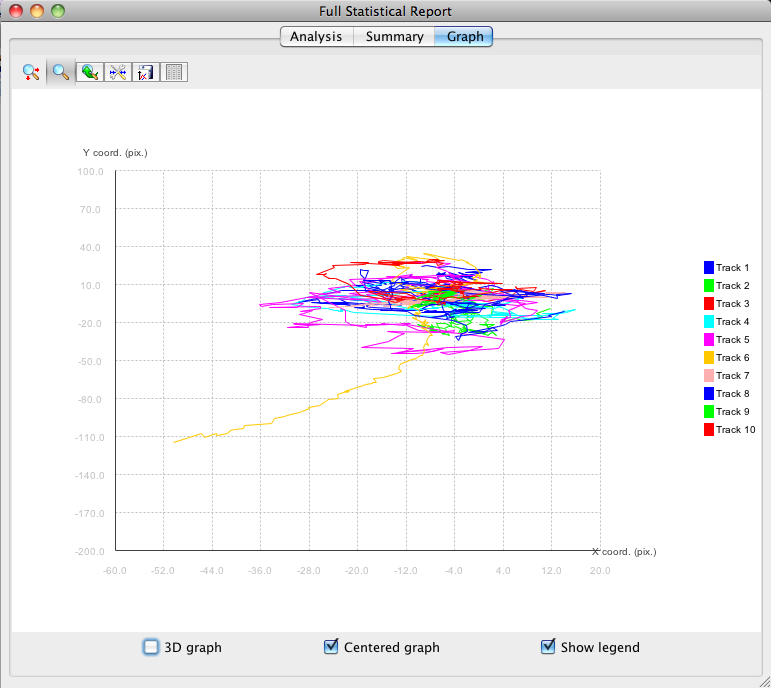


**Figure S16**: Centered Graph representation.

**Show legend:** If ticked, the legend is displayed.

*N.B. 1: Clicking on a track will highlight its legend.*

*N.B. 2: Double-clicking on a track will open the "Data" window.*

*N.B. 3: Double-clicking on a track name in the legend will open a new entry window where the name of the track can be changed.*

**Icons**: User may interact with the graph using the icons placed on the top left corner of the “Graph” tab.

**Center axes**

This allows the graph to be moved relative to the window.

**Zoom**

Use the mouse wheel to zoom the graph in and out.

**Rotate axes**

Only applies for 3D views; click and drag the graph to rotate it.

**Reset zoom & axes**

Restores the default view.

**Set scales**

Changes the titles of graph axes, the minimum and maximum bounds and the scale (linear or logarithmic).

**Save graphics in a .PNG file**

Saves the graph in a PNG (portable network graphic) file.

**Get data**

This opens a new "Data" window which lists the coordinates of the cell at each time point. By clicking on the button located on the right of "visible", one can change the color assigned to the selected track. There are also two icons in the window that allow the coordinates of the selected track to be saved either to the clipboard, or as an ASCII file.

### 4d3. “Delete cell” button

This will delete any chosen analyzed cell and its associated information.

## 4e. “Output” tab

The window is composed of two parts:

-“Visualisation” generates a copy of the active image, on which points or shapes of interest are drawn.

- “Save Data” is used to export numerical data in various formats (always as tab separated values that can be reimported into spreadsheet-based software).

### 4e1. Visualization

This visualizes the sectors, the mass centers of the kernels, the paths and the location of the center of the cells on each time frame image.

*N.B. There are two cases in which it is possible to superimpose tracks on to the original movie:*

*i) If tracking has just been done, the pre-processed image is still open. First close it and re-open the original image. A window should pop-up asking whether or not the current cells should be deleted. Click on “NO” and proceed to the visualization tab as mentioned.*

*ii) If tracking was performed previously the original image is still available as long as the XML file was saved. Re-open both and proceed to the visualization tab.*

#### 4e1a. “Sectors” button

This shows the inner and outer kernels of all tracked cells throughout the entire movie (Figure S17).


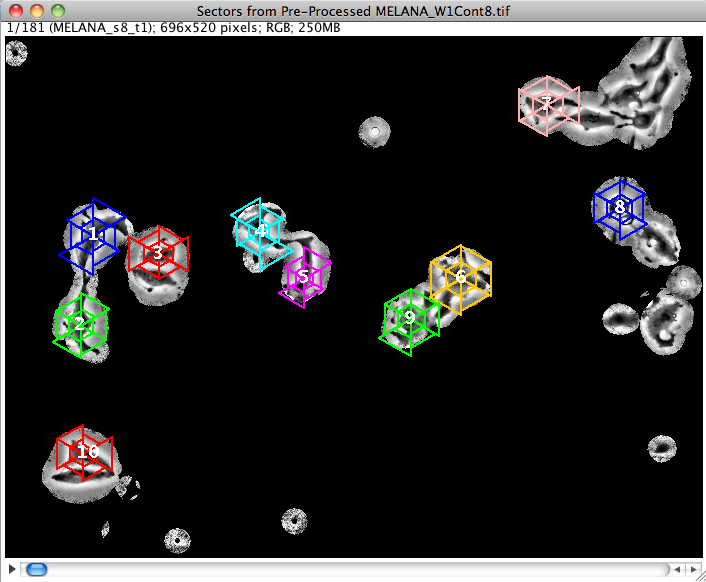


***Figure S17.*** *Superimposition of the 10 selected cells with their 12 kernels (6 inners & 6 outers) on each frame of the pre-processed movie. Here, the first picture of the movie is presented.*

#### 4e1b. “Centers” button

This shows the mass center (C) of all tracked cells throughout the movie (Figure S18).


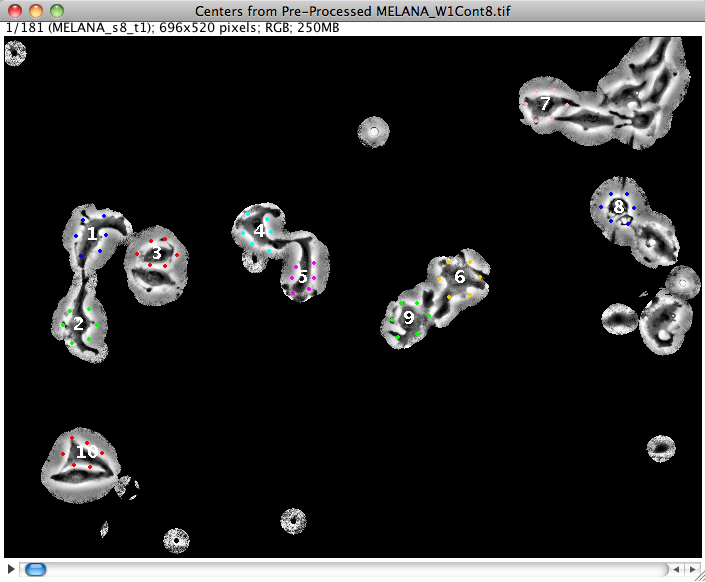


***Figure S18.*** *Superimposition of the 10 selected cells with the 6 mass centers on each frame of the pre-processed movie. Here, the first picture of the movie is presented.*

#### 4e1c. “Sectors and centers” button

This shows the inner and outer kernels, and respective mass centers (C), of all cells tracked throughout the movie (Figure S19).


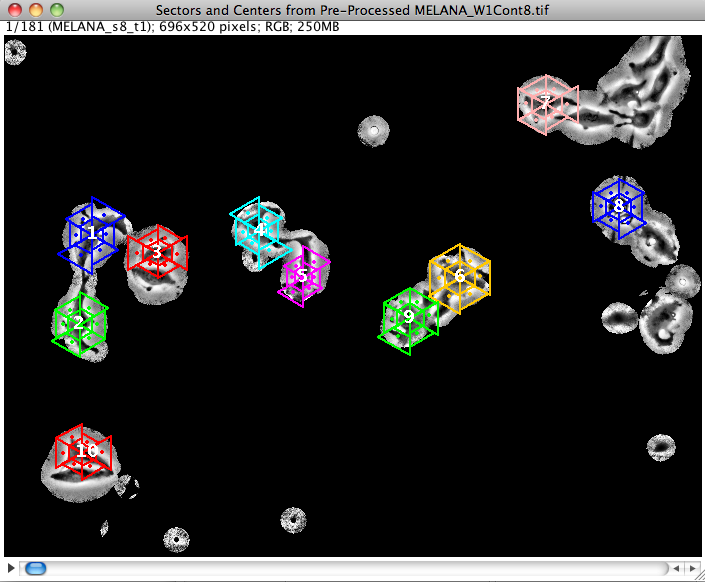


***Figure S19.*** *Superimposition of the 10 selected cells with their 12 kernels (inner & outer) and their associated mass centers on each frame of the pre-processed movie. Here, the first picture of the movie is presented.*

#### 4e1d. “Dots” button

Shows the centers of all cells tracked throughout the movie (Figure S20).


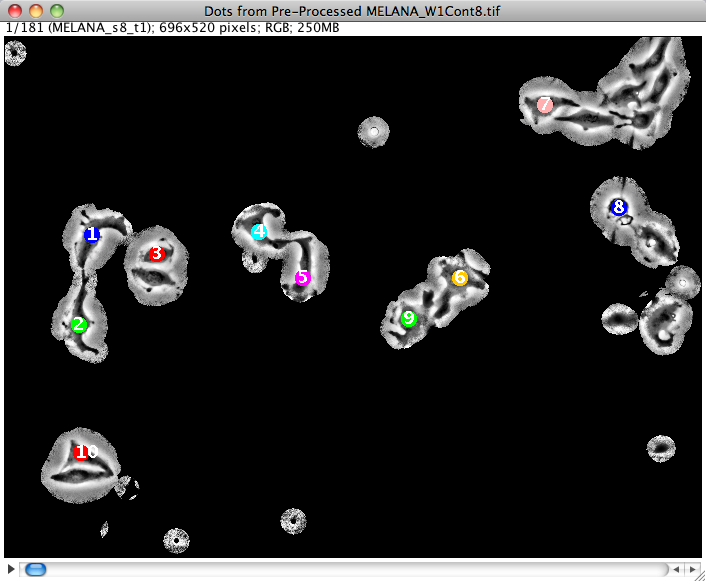


***Figure S20.*** *Superimposition of a dot representing the center of each cell on each frame of the pre-processed movie. Here, the first picture of the movie is presented.*

#### 4e1e. “Lines” button

Shows a line corresponding to the path of each cell tracked throughout the entire movie (Figure S21).


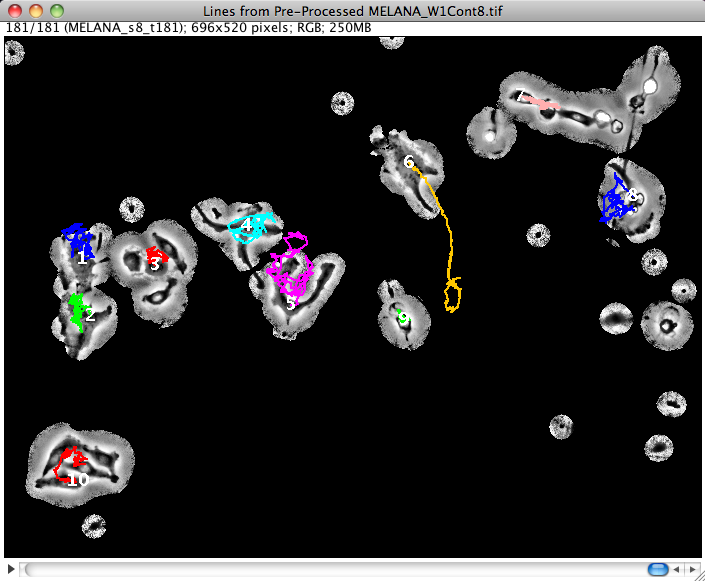


***Figure S21.*** *Superimposition of a line representing the path of each cell on each frame of the pre-processed movie. Here, the last picture of the movie is presented.*

#### 4e1f. “Dots and lines” button

Shows a line corresponding to the movement of the center, and the path, of each cell tracked throughout the movie (Figure S22).


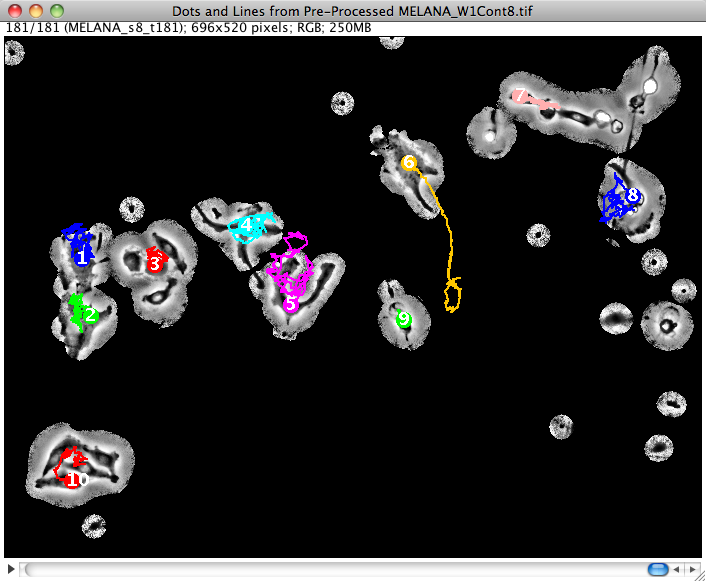


***Figure S22.*** *Dots and Lines are superimposed on the 10 selected cells. Their current locations are represented as colored dots and their paths are those from the full length of the pre-processed movie. Here, the last picture of the movie is shown.*

#### 4e1g. “Visu. Options” button

By clicking on the "Visu. Options" button, a new window will open (Figure S23). This enables modifications of the dot and line width and the font size used to display the cell number. All values are expressed as a number of pixels. Unticking “Draw Numbers” will stop the cell number from being displayed.


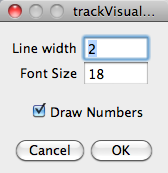


***Figure S23.*** *“trackVisual…” allows modifications of* *the dot and line width and font size used to display the cell number.*

### 4e2. “Save Data”

The “Save Data” function allows data to be saved as either tabulations separated values (XLS format) or as an XML file with the list of parameters that were used to pre-process/track/analyze and/or variables resulted from tracking/analysis.

#### 4e2a. “Save cells list” button

This saves an excel file (.xls) corresponding to the list generated in the "Setup" section. It includes the list of cell numbers with their original coordinates (x_0_, y_0_), the total time interval of the movie (number of frames - 1), nDir, Rad, RbOnRw (=Rb/Rw), kappa, beta, minRad and maxRad.

#### 4e2b. “Save tracks list” button

This saves an excel file (.xls) corresponding to the list generated in the "Tracking" section. It includes the list of all successive coordinates (x,y) of each tracked cells for each time point (t) in minutes.

#### 4e2c. “Save MT style” button

This option allows data to be exported as an excel file (.xls) in the same format as those used in the Manual Tracking Plugin for ImageJ (<http://rsbweb.nih.gov/ij/plugins/track/track.html>). It includes “Track ni” (the cell number), “Slice ni” (the frame number), X and Y (the coordinates of the cells), “Distance” (the Euclidian distance (or instantaneous distance) between two consecutive time points) and “Velocity” (the instantaneous speed). The “Pixel Value” is required for import to Manual Tracking and is not filled by iTrack4U.

#### 4e2d. “Save analysis” button

This option allows the list of instantaneous information for each cell to be saved in an excel file (.xls). Data includes the instantaneous distance (μm), speed (μm/min), acceleration (μm/min^2^), the angle (°) and the delta angle (°); all variable descriptions might be found in paragraph 4d2.

#### 4e2e. “Save summary” button

This list of information generated from the full statistical report (see above) as an excel file (.xls).

#### 4e2f. “Save image” button

This option enables the active image to be saved (as a .tif file).

#### 4e2g. “Export to XML” button

This option saves the full analysis as two files:

- XML file (named after the dataset): this file contains all parameters used for pre-processing, tracking and analysis together with the data. Each item is tag-delimited for further reuse with external software.

- XSL file (full-report.xsl): as the volume of data within the XML file may be huge and difficult to read, a style sheet can be applied to the XML file to make it accessible from a web browser. This file is used when double-clicking on the XML file (*N.B*. For the style sheet to be used, XSL and XML files should be stored in the same folder).
