# Supplementary figures and images for: Automated Cell Tracking and Analysis in Phase-Contrast Videos (iTrack4U): Development of Java Software Based on Combined Mean-Shift Processes
Source: PLoS One. 2013 Nov 27;8(11):e81266. doi: 10.1371/journal.pone.0081266 (PMC3842324; doi:10.1371/journal.pone.0081266)

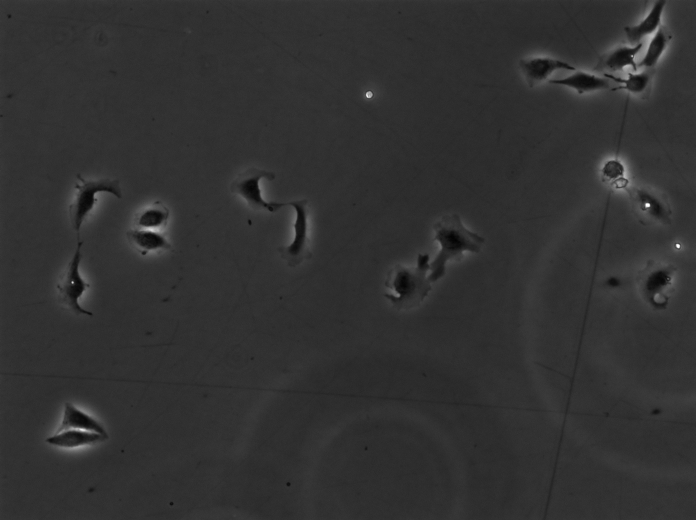

Supplement: Movie S1 — Stack of images. (ZIP) [file pone.0081266.s003.zip › MELANA_W1Cont8.tif]

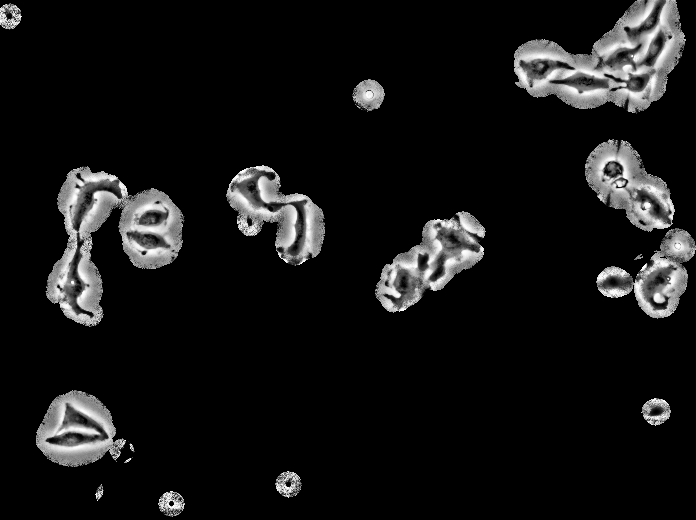

Supplement: Movie S2 — Preprocessed movie. (ZIP) [file pone.0081266.s004.zip › movie S2.tif]
